# Alternative Procedure to Individual Nasal Pressure Titration for Sleep Apnea

**DOI:** 10.3390/jcm10071453

**Published:** 2021-04-01

**Authors:** Ramon Farré, David Gozal, Josep M. Montserrat

**Affiliations:** 1 Unitat de Biofísica i Bioenginyeria, Facultat de Medicina i Ciències de la Salut, Universitat de Barcelona, 08036 Barcelona, Spain; 2 CIBER de Enfermedades Respiratorias, 28029 Madrid, Spain; jmmontaserrat@ub.edu; 3 Institut Investigacions Biomediques August Pi Sunyer, 08036 Barcelona, Spain; 4 Department of Child Health, The University of Missouri School of Medicine, Columbia, MO 65212, USA; gozald@health.missouri.edu; 5 Sleep Lab, Hospital Clinic, Universitat de Barcelona, 09036 Barcelona, Spain

**Keywords:** sleep apnea, CPAP treatment, titration

## Abstract

In the treatment of obstructive sleep apnea (OSA), the current standard of “CPAP titration” in the laboratory or at home is a resource demanding and costly approach that, in developed economies, markedly augments healthcare costs and in low resource economies precludes access to care altogether. Here, we discuss that current guidelines for titration of CPAP could be obviated by taking a different route that in many ways is similar to the institution of treatment in many other medical conditions. To this effect, we present novel population based data from 16,780 patients, showing that after individualized and labor-intensive and expensive CPAP titration, 86.4% of OSA patients are treated with nasal pressure settings within the range of 9 ± 2 cmH_2_O, and review the literature to justify the potential adoption of a standard therapeutic CPAP setting as the initial intervention which would be subsequently followed by any necessary adjustments in only a minority of patients who would not derive the necessary benefit from such standardized intervention. Assuming an 80–85% success rate as derived from our analyses, our personal view if extensively adopted could radically reduce healthcare costs and enable markedly improve access to diagnostics.

## 1. Introduction

Continuous positive airway pressure (CPAP) is the most effective and widely used treatment for obstructive sleep apnea (OSA) [1]. In clinical practice it is common that after OSA is diagnosed and before CPAP is prescribed, a personalized CPAP titration is carried out to determine what nasal pressure settings and device mode are required to treat each patient [2]. It is, however, noteworthy that CPAP is among the few long-term therapies for chronic diseases in which the “dose” prescribed to each patient is titrated before the actual initiation of the treatment. Indeed, most common drugs in adults are usually prescribed using a standard dosage regardless of sex, age, weight, or any other individual characteristics. It is only when such standard dosage fails to achieve the desired therapeutic effects that the initial dosage is modified, usually in a trial-and-error empirical manner.

The reason why CPAP is individually titrated before implementation is probably historical. Indeed, CPAP is a relatively recent treatment modality that was initially conceived in sleep physiology research laboratories [3] and, as the evidence confirming its positive therapeutic effect began to emerge, it was progressively translated from research labs to patients [4]. At the time of CPAP discovery, the number of patients was quite low, since awareness of OSA was barely developing, and as such, personalized CPAP titration did not pose major problems. Nowadays, in light of the considerable number of patients who suffer from OSA [5,6], individual CPAP titration has become a major logistic and financial burden [7]. Fortunately, CPAP titration can be simplified by approaches that do not require full in-hospital polysomnography (PSG), such as relying on respiratory polygraphy or using automatic CPAP devices at home. Nevertheless, even when employing these simplified approaches, the burden of CPAP titration remains considerable for health care systems [7]. Remarkably, this problem will continue to escalate in the future with the global epidemic of obesity [8] and the increased ageing of the population, both of which are well known to enhance OSA prevalence and severity [9,10]. This difficult situation is and will be further exacerbated in regions with reduced resources, where the prevalence of OSA is increasing [6,11] and an institution of the conventional procedures normally used for personalized CPAP titration is virtually impossible to achieve due to the cost of the equipment, the challenging logistics, and the paucity of professional expertise.

## 2. Alternative Proposal to CPAP Titration

Given that individually titrating CPAP is not a life-threatening issue and is not of critical importance to achieve a radical improvement in the patient’s health, it is reasonable to think about alternative simplified procedures leading to similar therapeutic effectiveness. Hence, based on the new data presented herein and based on conceptual assumptions, we propose that in case that personalized CPAP titration may impede or substantially delay treatment, CPAP should be initially prescribed at 9 cmH_2_O to all recently diagnosed OSA patients who would normally be eligible for this treatment, and that in case the patient still manifests residual symptoms, then a visit to the healthcare staff would be needed to modify the CPAP settings. To further reduce the burden to health systems, particularly in low-resource settings, it could also be possible to ask the patient to adjust the CPAP setting (within a safety preset range) depending on symptoms and comfort. In fact, such a patient-empowering procedure has proven its worth in a seminal study that surprisingly has seen no continuation and further validation despite the promising results [12].

## 3. Data on Conventionally Prescribed CPAP in OSA

**Figure 1:**
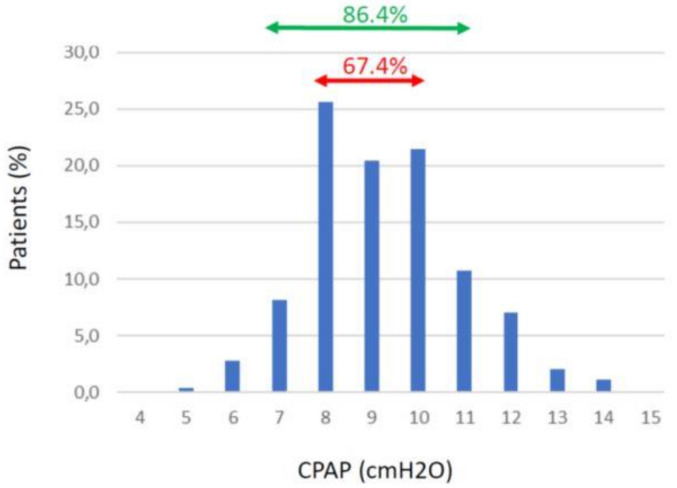
Percentage of patients routinely treated with different CPAP pressure settings among 16,780 unselected patients suffering from OSA in Catalonia, Spain.

Analysis of the actual distribution of CPAP pressure settings in clinical practice provides the required justification for the proposed alternative procedure. Figure 1 shows information usually not provided in the literature and consists of the histogram of the actual pressure settings for nasal CPAP retained for treating 16,780 unselected patients who underwent conventional CPAP titration in the area of Catalonia, Spain. These data reveal that the mean (±SD) of CPAP pressures retained for the treatment of this large cohort of patients was 9.3 ± 1.7 cmH_2_O, and constitute pressure settings that are remarkably similar to those reported in many research studies involving smaller numbers of patients who were selected/excluded according to specific criteria [13,14]. Most interestingly, the real-life data in Figure 1 indicate that 67.4% of patients are treated with CPAP values within a range of 8–10 cmH_2_O, i.e., differing by only ±1 cmH_2_O from the mean of 9 cmH_2_O. Moreover, in an additional 19.0% of patients—i.e., those treated with CPAP settings of 7 (8.2%) or 11 cmH_2_O (10.8%)—a CPAP pressure of 9 cmH_2_O would represent a difference of only ±2 cmH_2_O from the pressure settings selected through personalized titration. Thus, after individualized and labor-intensive and expensive CPAP titration, 86.4% of OSA patients are treated with nasal pressure settings within the range of 9 ± 2 cmH_2_O.

## 4. Discussion

### 4.1. Optimal CPAP Obtained by Conventional Titration: An Elusive Concept

**Figure 2:**
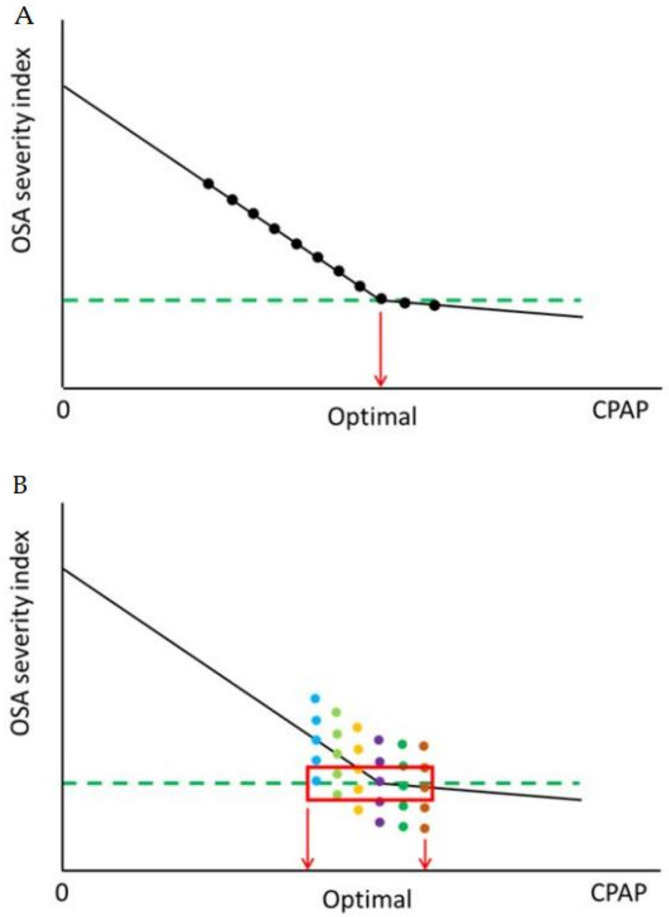
CPAP titration procedure. (**A**): Conventional rationale for titrating optimal CPAP pressure settings. The obstructive sleep apnea (OSA) severity index is reduced as CPAP is increased (black dots) until reaching an accepted threshold (e.g., 5 events/h) considered the optimal CPAP pressure setting for treating the patient (green dotted line). (**B**): Schematic diagram illustrating how the OSA severity index (different dot colors) for each CPAP pressure varies along different testing nights. This variability results in a window of potential “optimal” CPAP pressure settings (red box).

In favor of our simplifying proposal, it is also pertinent to mention that the concept of optimal CPAP for a given patient is unclear [15]. The top panel in Figure 2 represents the theoretical rationale behind the conventional concept of individualized CPAP titration: the respiratory events experienced by untreated OSA patients are progressively reduced as CPAP pressures are incrementally raised until reaching a point where the number of residual events is below a predefined value considered as acceptably close to normal (e.g., <5 events/h), and beyond this threshold value (which is open to debate [16]) further increase in CPAP results in minor or no further reductions in the number of events. This model accurately reflects the relationship between the number of events and CPAP pressures during a single night. However, a first conceptual question is to what extent CPAP must eliminate all the respiratory events, including the mildest ones, such as snoring and flow limitation events, which are frequent events in the normal population. This issue may alone represent an increment of 1.5 cmH_2_O mean difference in optimal CPAP settings [17]. Another issue is how robust and reliable the pressure setting obtained from the titration of any given patient is, and whether the concept of optimal CPAP is consistently applicable in the real clinical arena. Whereas a considerable amount of clinical research has been devoted to compare different methods to titrate CPAP―including PSG, respiratory polygraphy, automatic CPAP devices and predictive formulas― [18,19,20,21,22], there is scant convincing information available on the reliability and validity of titration. For instance, Wiest and coworkers titrated CPAP for two consecutive nights in OSA patients and reported a small, but statistically significant difference between the two sessions. More importantly, they found that for 50% of patients the difference between the two titration sessions was within ±3 cmH_2_O (or 2–3 cmH_2_O in 34% of patients) [23].

The difficulty of determining an optimal CPAP value from the information gathered during a conventional one-night titration emerges more clearly from a recent study by Callaghan and coworkers who systematically evaluated the number of residual respiratory events when 7 different pressure settings varying between 2 and 3 cmH_2_O below and 1–2 cmH_2_O above the “optimal” prescribed pressure setting (as determined by a full laboratory titration) were applied for 15.9 ± 5.1 nights in 28 OSA patients (24). These investigators documented that application of a given CPAP value resulted in a different number of events across nights, as schematically illustrated in the bottom panel of Figure 2. Accordingly, in a considerable proportion of OSA patients treated with CPAP being titrated on a single night (conventional titration procedure), the final nasal pressure setting may be within ±2–3 cmH_2_O from the optimal setting that would result from a multi-night titration. Consequently, Callaghan et al. concluded that the best way to determine the CPAP pressure setting for any given patient would require the derivation of a pressure value calculated by data analysis from a titration procedure consisting in of multiple nights. Whereas such a cumbersome approach could result in a more robust and valid CPAP setting value, the implementation of this methodology would be prohibitively expensive and require specific equipment incorporating advanced algorithms [24], particularly in some healthcare system settings under strained financial conditions. Whereas different home auto-CPAP titration approaches [25] or auto-CPAP treatment, including the use of telemedicine [26], are potential solutions to address intra-subject variability regarding optimal nasal pressure requirements, these solutions are not widely applicable or implementable because there are incremental costs associated with these devices, which are not always covered by healthcare insurance providers, and therefore, many years after auto-CPAP appearance in the marketplace, this approach, while used in certain countries, is not universally adopted even in high income countries including Europe and the U.S. In this regard, it this remarkable that a considerable number of studies comparing different methods of CPAP titration have shown that applying nasal pressure settings that differ by around 2 cmH_2_O on average will have no impact on the clinical effectiveness of the treatment in terms of reduction of respiratory events and somnolence [19,20,21,22]. Consequently, these data give further support to our proposal of applying an initial standard pressure setting value (9 cmH_2_O) and that such approach should be clinically effective in a vast majority of OSA patients, albeit not necessarily ideal.

### 4.2. Potential Drawbacks of the Proposed Approach

Treating some patients with a pressure clearly below optimal value could translate into the presence of residual events (snoring, flow limitation, or mild hypopneas) and a potentially less favorable mitigation of symptoms, and thus adherence, or not fully reducing the risk of cardiovascular consequences of OSA. However, most detrimental events such as apneas and severe hypopneas would disappear. Conversely, those patients receiving a CPAP pressure that is higher than the individually derived optimal one would be “overtreated” with a pressure setting of 9 cmH_2_O. A nasal pressure that is set a few cmH_2_O above the ideal one is unlikely to be detrimental (e.g., for cardiac output), and would scarcely impact on patient comfort and thus adherence. More importantly, in this situation, all the patient’s respiratory events would be eliminated. We should emphasize that the potential drawbacks resulting from deviating from “ideal” CPAP by ±2–3 cmH_2_O would be certainly acceptable if the alternative was no CPAP treatment because of the lack of financial resources or logistical infrastructure permitting implementation of the conventional CPAP titration. Clearly, the proposed setting of 9 cmH_2_O is not written in stone. Although it seems suitable for covering 86.4% of patients (within a ±2 cmH_2_O range) in a general population of OSA patients (Figure 1), the initial CPAP figure could be moved to 10 cmH_2_O to reduce potential residual effects in some additional patients. It also could be further increased in specific subsets of patients in whom previous experience and their characteristics would predict that they require higher CPAP therapeutic pressures (e.g., in morbid obesity or when using a facial mask [27]).

### 4.3. Potential Applicability of the Proposed Approach

The approach we propose here consisting of simply eliminating the step of individual CPAP titration as currently performed is a pragmatic application of the classical aphorism that “perfect is the enemy of the good”. It may seem a drastic departure from the daily clinical routine. However, to enhance further the attractiveness of this proposal, we could hypothesize the situation whereby CPAP treatment for OSA would be a recently developed medical therapy. It seems clear for us that, considering the data provided in Figure 1 (85% patients satisfactorily treated with an initial value of CPAP around 9 cmH_2_O) healthcare regulatory bodies would recommend our proposed approach instead of adopting a complex and expensive procedure as the current one based on systematic overnight CPAP titration in all patients.

Regarding recommendations from medical societies, it is worth noting that, in a real world setting, professional guidelines will need, along with the publication of recommendations for treatment of OSA based on the very best possible procedures that are potentially applicable to health care systems with optimal human and material infrastructure, to address the financial capability to implement such recommendations, as well as include effective and safe contingency plan recommendations that are suitable alternatives when operating in different socio-economic and infrastructure environments. Such an adaptive set of guidelines would then enable clinicians to operationalize their approach at the individual patient level.

Interestingly, the financial resources and professional efforts eventually spared by avoiding CPAP titration in most OSA patients (only a minority of complex patients or those situated in the edges of the Gaussian distribution shown in Figure 1 would require individualized assessment after initial CPAP trial) could be reinvested into OSA patients; for instance, to expand the access to diagnose the disease, which is also a costly procedure [7], or to implement programs aimed at improving CPAP patient’s adherence [28,29].

## 5. Conclusions

In light of the previous reasoning, testing our proposal appears worthy of future clinical trials to substantiate its applicability. We acknowledge that the approach we postulate herein might prove rather disruptive and interfere with established daily routines and conventional mindsets. However, the current COVID-19 pandemic has shown to the medical community that some of the well-stablished routine clinical protocols can be actually changed without detrimental effects and in some cases may lead to improved clinical outcomes.

## Data Availability

No patient data were used. The study is exclusively based on de-identified data from CPAP devices (pressure setting).
